# Gender and Age Effects on the Trajectory of Depression in Opioid Users during Methadone Maintenance Treatment

**DOI:** 10.3389/fpsyt.2017.00288

**Published:** 2017-12-13

**Authors:** Peng-Wei Wang, Huang-Chi Lin, Yi-Hsin Connie Yang, Chih-Yao Hsu, Kuan-Sheng Chung, Hung-Chi Wu, Cheng-Fang Yen

**Affiliations:** ^1^Department of Psychiatry, Kaohsiung Medical University Hospital, Kaohsiung, Taiwan; ^2^Department of Psychiatry, Faculty of Medicine, College of Medicine, Graduate Institute of Medicine, Kaohsiung Medical University, Kaohsiung, Taiwan; ^3^Department of Pharmacy, Kaohsiung Medical University, Kaohsiung, Taiwan; ^4^Department of Addiction Science, Kai-Suan Psychiatric Hospital, Kaohsiung, Taiwan; ^5^Department of Community Science, Kai-Suan Psychiatric Hospital, Kaohsiung, Taiwan

**Keywords:** opioid, methadone, depression, trajectory, gender, age, addiction

## Abstract

**Introduction:**

Both heroin use and depression are significant health problems. Methadone maintenance treatment (MMT) can be of great benefit to heroin users. However, changes in the level of depression in heroin users during MMT are not clear. Gender and age are also important factors in the development of depression, and whether gender and age moderate changes in depression in heroin users during MMT warrants further study. This study aimed to explore: (1) the trajectory of depression in opioid users during MMT and (2) the moderating effects of gender and age on the trajectory of depression in opioid users receiving MMT.

**Method:**

A total of 294 intravenous heroin users were recruited into this 9-month observational study. The level of depression was measured at the intake interview and at follow-up interviews every 3 months. A latent growth model was used to analyze the trajectory of the level of depression among the participants.

**Results:**

Depression improved rapidly during the first 3 months of MMT and slowly after the first 3 months in both the female and male heroin users. There was no gender difference in the level of depression at each follow-up point. The level of depression in the female heroin users decreased faster than that in the male heroin users. In addition, the level of depression in the younger heroin users decreased faster than that in the older subjects.

**Conclusion:**

Depression in female and younger heroin users improved more rapidly than in male and older subjects, respectively.

## Introduction

Heroin dependence has many negative effects, such as inadvertent overdose, increased risk of infection, unemployment, social cost due to criminal activities, and family disruption (1). Around 0.7% of adults worldwide use opioids, the most common of which is heroin (2). Therefore, heroin use disorder is an important health issue.

Depression is a commonly occurring, serious, recurrent disorder linked to diminished role function and quality of life, medical morbidity, and mortality (3). Depression was ranked the fourth leading cause of disability worldwide by the World Health Organization and is expected to be the second leading cause in 2020 (4). In addition, a previous study demonstrated that substance use disorder is positively associated with depression (5). The prevalence of depression among heroin users is high, ranging between 20 and 45% (6, 7). Furthermore, heroin users with depression may be associated with greater drug use and risky behavior (6). Therefore, depression is an important mental issue in opioid users.

Methadone maintenance treatment (MMT), an opioid agonist, has been used to treat heroin users for heroin dependence since the mid-1960s (8). MMT contributes to decreases in drug use, drug-injecting behaviors, drug-related criminal behaviors, unprotected sexual behaviors, human immunodeficiency virus infections, and overdose deaths, and improves psychiatric comorbidity (9–12). Previous studies have indicated that the rate of current major depression declined by 15% during 12-month MMT, and the rate of clinically significant depression decreased by 30% during 18-month MMT (6, 13). In the general population, the trajectory of the course of depressive symptoms is quite heterogeneous, which is important for depression treatment (14); however, the trajectory of the decline in the level of depression during MMT is not clear, and is an important question in patients with MMT.

Depression is more prevalent among women than men, the male to female ratio being approximately 1:2 (15). A study of alcohol users found that female alcohol users had a higher level of depression than males (16). Regarding heroin users, women may be more likely than men to suffer from all kinds of mental illness (17). Both studies supported the idea that female heroin users may suffer more depressive problems than male heroin users, regardless of age. Furthermore, a study indicated that depression was more prevalent among female than male regular heroin users (18). All these results indicated that gender is important in relation to heroin users’ mental health, especially depression. Therefore, the way in which gender moderates change in the level of depression in opioid users undergoing MMT is an important issue.

In the general population, age plays an important role in the severity of depression, the level of depression increasing with age (19). In opioid users, cross-sectional study showed that age is a risk factor for depression (7). Therefore, the way in which age moderates the trajectory of depression is important for clinicians. Latent growth curve modeling (LGM) is an application of a structure equation model (SEM) used to analyze the change of repeated measures of a variable (20). One of the advantages of LGM is the ability to model aspects of change, and not only to describe a single individual trajectory, but also to capture individual differences in trajectories over time. Another advantage of LGM is its flexibility in allowing researchers to fully examine the questions that they propose. In this study, LGM was used to study the trajectory of the level of depression in heroin users undergoing MMT. The aims of the present study were to explore: (1) the trajectory of depression in opioid users during 9-month MMT and (2) the moderating effects of gender and age on the trajectory of depression in opioid users receiving MMT.

## Materials and Methods

### Research Design and Participants

This 9-month prospective observational study, conducted from August 2013 to July 2015, recruited consecutive intravenous heroin users with heroin dependence according to the criteria of the DSM-IV-TR (21). All participants were referred by their doctor when they visited outpatient methadone clinics of three hospitals (two general hospitals and one psychiatric hospital) in southern Taiwan. Psychiatrists used the Mini International Neuropsychiatry Interview to determine participants’ psychopathologies. Mental state was determined from each participant’s past history. Those who had severe psychopathologies such as schizophrenia, bipolar disorder, or major depressive disorder, mental retardation, or those who had severe heroin withdrawal symptoms that might inhibit their ability to comprehend the procedure and questionnaires, were excluded. Three-hundred sixty patients were screened, and 66 refused to participate. A total of 294 intravenous heroin users agreed to participate and completed this study. None of the participants had received MMT before the interview at intake or took any psychotropic drugs during the study. During the 9-month follow-up period, participants were re-evaluated by research assistants, blind to the baseline data, every 3 months. The protocol was approved by the Institutional Review Board of Kaohsiung Medical University. Written informed consent was obtained from all participants prior to entry into the study. All participants received 10 USD for compensation when they completed the study.

### Survey Instruments

The participants underwent interviews at intake to collect baseline data, including age, sex, and level of depression, before receiving MMT. At the follow-up interviews, the level of depression as measured using the Taiwanese version of the Center for Epidemiological Studies Depression Scale (CES-D), and the mean dosage of methadone during the past 3 months was recorded. The Taiwanese version of the CES-D has been used for the study of depression in Taiwan for many years (22). Cronbach’s alpha for the CES-D was 0.875. The total score on the CES-D ranged from 0 to 60, with higher scores indicating more severe depressive symptoms. Meanwhile, we determined a total score of 15 to be the cutoff point for clinically significant depression based on previous studies (13, 22).

### Data Collection Procedures and Statistical Analysis

**Figure 1:**
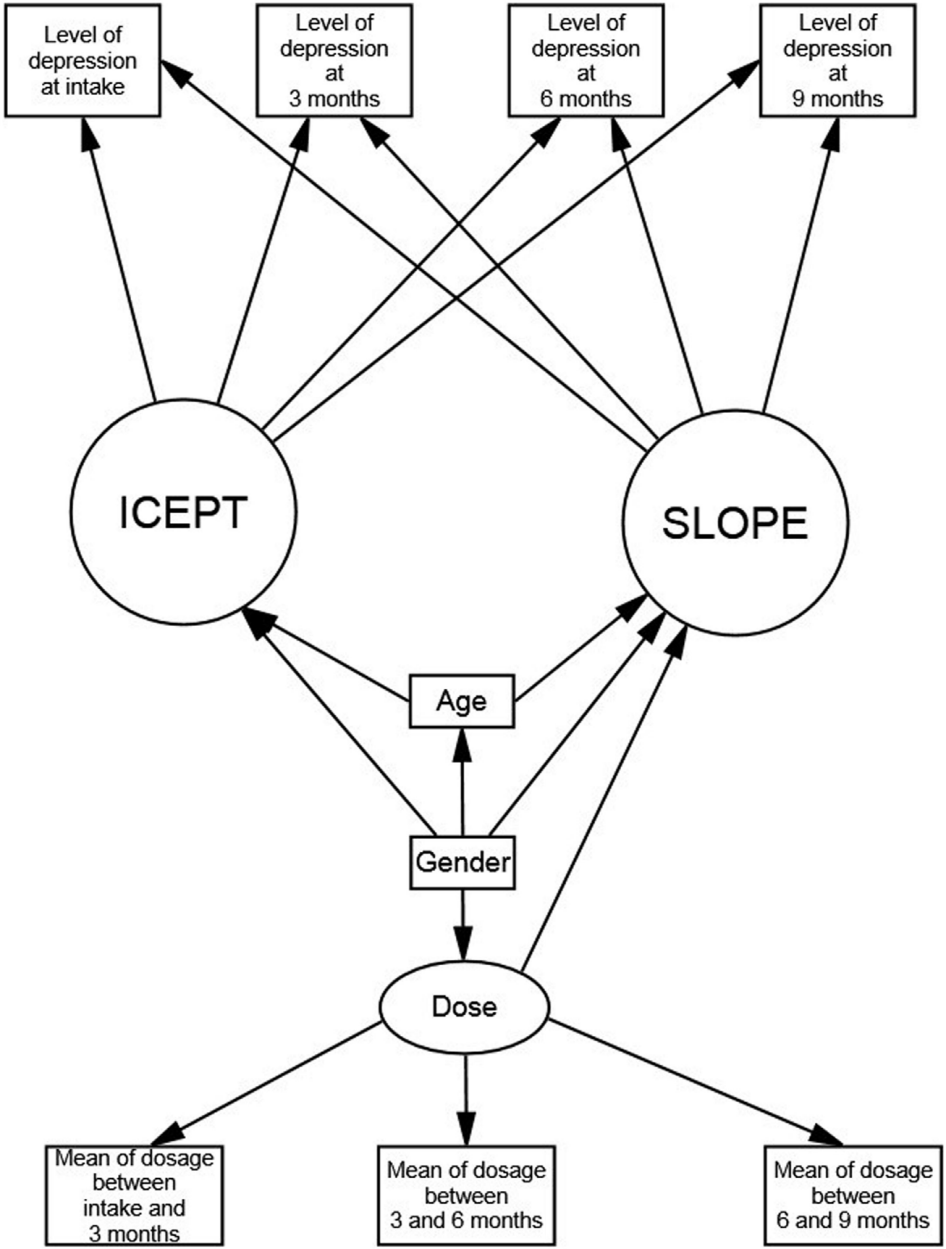
Latent growth model of the level of depression in heroin users undergoing methadone maintenance treatment.

Data analysis was performed using SPSS 17.0 software (SPSS, Chicago, IL, USA). We used descriptive statistics, including means and SDs, to describe socio-demographic data, methadone dosage, and level of depression. Baseline characteristics were compared between the male and female heroin users using Student’s *t*-test. ANCOVA was used to examine the gender differences in the level of depression and dosage of methadone, controlling age, and education. Cohen’s *d* was used to calculate effect sizes. To examine the difference in the level of depression between intake and each follow-up point, paired *t*-tests with adjusted multiple comparisons were used. Then, we performed LGM in the SEM using Analysis of Moment Structures 18 to explore how the level of depression changed in the heroin users during the course of the MMT program. The hypothesized model was as shown in Figure 1. The indices used for estimating the goodness of fit of the model were the χ^2^ goodness of fit value (*p*-value) >0.05, the comparison of fit index (CFI) >0.9, and the root mean square error of approximation (RMSEA) <0.1. We made inferences at the 0.05 level of significance for all inferential statistical procedures.

## Results

The epidemiological characteristics, level of depression, and dosage of methadone at each hospital are shown in Table 1. These data did not differ significantly among the three hospitals. The level of depression and dosage of methadone at each follow-up point are shown in Table 2. The mean of the level of depression at intake was significantly higher than a CES-D score of 15 (*t* = 8.44, *p* < 0.001), indicating a clinically significant level of depression. The level of depression after 3, 6, and 9 months was significantly lower than that at intake (paired *t* = 8.61, 8.66, and 9.76, respectively, *p* < 0.001 for all three). There was no significant difference in the level of depression at the end of 3, 6, and 9 months (paired *t* = 0.58, 1.13, and 1.79, respectively, *p* = 0.561, 0.222, and 0.061, respectively). There was no significant difference in the dosage of methadone between the first, second, and third 3-month periods (paired *t* = −1.11, 1.39, and 0.55, respectively, *p* = 0.267, 0.165, and 0.583, respectively).

**Table 1:** Epidemiology, level of depression, and dosage of methadone among the three hospitals.

	*N* (%) (*n* = 69), general hospital I	*N* (%) (*n* = 69), general hospital II	*N* (%) (*n* = 156), psychiatric hospital	*p*-Value^a^
Gender (male)	64 (92.75)	61 (88.41)	132 (84.62)	0.228
	**Mean (SD)**	**Mean (SD)**	**Mean (SD)**	
Age (years)	38.3 (7.63)	39.96 (7.47)	38.76 (7.74)	0.534
Education (years)	9.78 (1.95)	9.74 (2.54)	10.12 (2.13)	0.382
**Level of depression**				
At intake	24.37 (12.94)	21.42 (11.32)	20.99 (11.02)	0.315
At end of the first 3 months	18.01 (11.75)	16.06 (9.70)	15.61 (9.59)	0.263
At end of the second 3 months	17.71 (12.52)	14.72 (10.67)	15.63 (10.22)	0.254
At end of the third 3 months	16.96 (11.66)	14.72 (10.46)	14.90 (10.19)	0.352
**Dosage of methadone (mg)**				
First 3-month period	50.07 (24.53)	53.84 (28.65)	48.93 (33.32)	0.536
Second 3-month period	58.48 (36.32)	61.96 (36.30)	55.16 (27.42)	0.326
Third 3-month period	56.96 (29.87)	59.86 (32.28)	53.85 (26.46)	0.335

*^a^χ^2^ test for categorical variables; ANOVA for other variables*.

**Table 2:** Level of depression and dosage of methadone at each follow-up point for heroin users.

	Total participants, mean (SD) (*n* = 294)	Male participants, mean (SD) (*n* = 257)	Female participants, mean (SD) (*n* = 37)	Effect size^a^	*F*-value^b^	*p*-Value^b^
**Level of depression**						
At intake	21.76 (11.54)	20.91 (11.29)	28.83 (11.74)	0.69	14.825	<0.001
At end of the first 3 months	16.29 (10.31)	16.00 (10.12)	18.43 (10.53)	0.24	3.355	0.068
At end of the second 3 months	15.91 (10.93)	15.60 (10.91)	18.54 (11.07)	0.30	3.849	0.051
At end of the third 3 months	15.34 (10.75)	14.82 (10.67)	18.62 (9.69)	0.40	7.957	0.005
**Dosage of methadone (mg)**						
First 3-month period	56.25 (29.24)	55.80 (28.86)	62.57 (31.52)	0.23	1.936	0.165
Second 3-month period	57.55 (31.97)	57.12 (31.89)	60.54 (32.83)	0.11	0.486	0.486
Third 3-month period	55.99 (28.73)	55.95 (28.84)	56.22 (28.34)	0.01	0.004	0.947

*^a^Cohen’s d*.

*^b^Controlling age and education*.

The mean age of the male and female heroin users was 39.62 (SD = 7.45) and 35.15 years (SD = 7.89), respectively. The gender difference for age was significant (*t* = −3.390, *p* = 0.001). Compared with the level of depression at intake, the male heroin users showed significant decreases at the end of 3, 6, and 9 months (paired *t* = 7.48, 7.37, and 8.45, respectively, *p* < 0.001, <0.001, and <0.001, respectively). However, the level of depression of the male heroin users after 3 months did not differ from that after 6 months (paired *t* = 0.67, *p* = 0.501), and the difference in the level of depression between the end of the 6- and 9-month periods was not significant (paired *t* = 1.35, *p* = 0.179). The methadone dosage of the male heroin users also did not differ significantly between the first, second, and third 3-month periods (paired *t* = −1.57, −0.13, and 1.04, respectively, *p* = 0.119, 0.898, and 0.299, respectively). Furthermore, the female heroin users also exhibited a significantly lower level of depression at the 3-, 6-, and 9-month follow-up points than at intake (paired *t* = 4.55, 4.93, and 5.33, respectively, all *p* < 0.001). No significant difference in the level of depression was found between the 3-, 6-, and 9-month follow-up points in the female heroin users (paired *t* = −0.21, −0.46, and −0.24, respectively, *p* = 0.835, 0.647, and 0.614, respectively). Meanwhile, the methadone dosage of the female heroin users did not change significantly between the first, second, and third 3-month periods (paired *t* = 0.77, 1.43, and 0.99, respectively, *p* = 0.449, 0.161, and 0.330, respectively).

**Figure 2:**
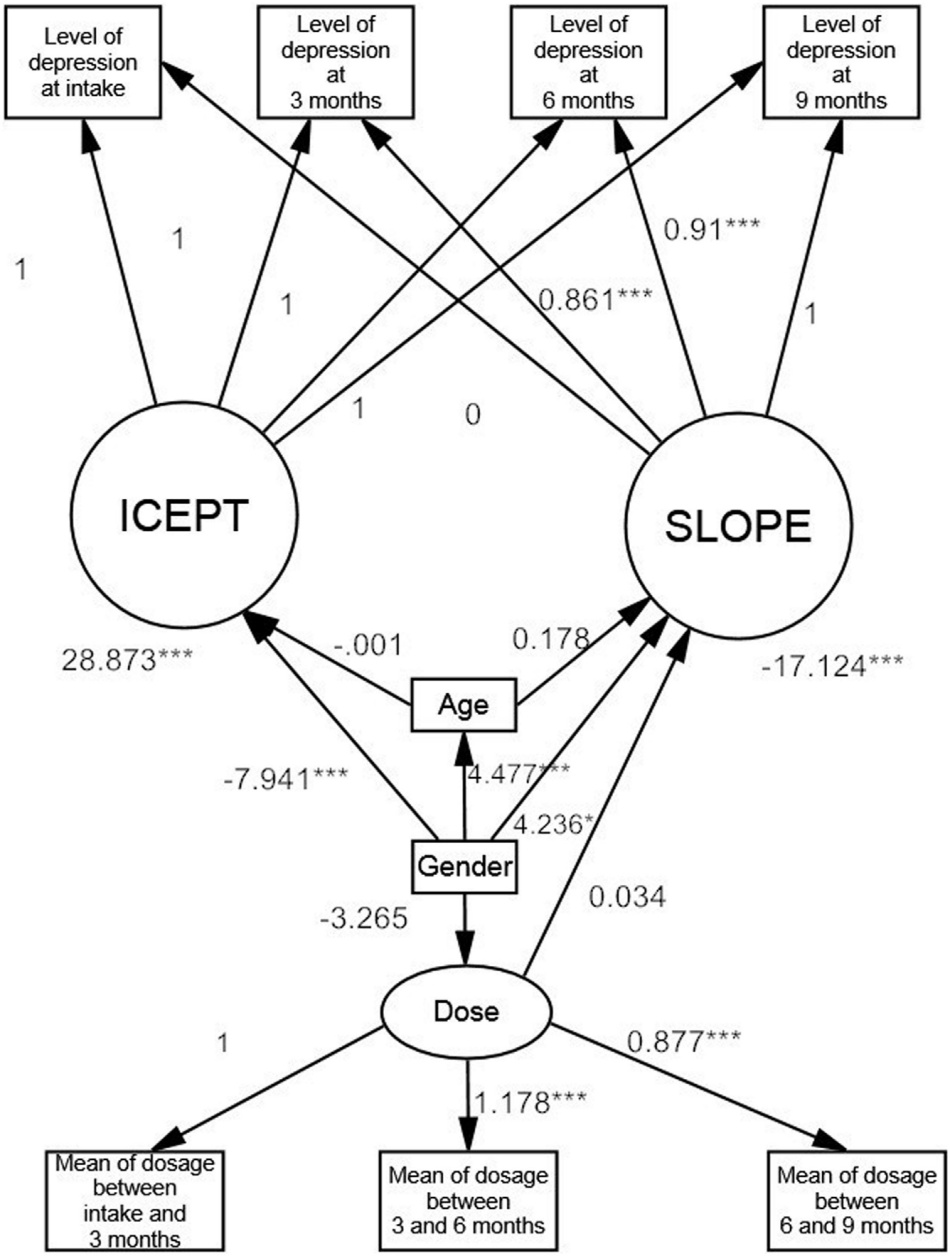
Moderating effects on the trajectory of depression in heroin users according to the latent growth model. *<0.05; **<0.01; ***<0.001.

The latent growth model is shown in Figure 2. The χ^2^ goodness of fit value (*p*-value) was 0.133, the CFI was 0.994, and the RMSEA was 0.033. All these indices showed that the latent growth model was of a good fit. The estimated mean of the slope (SLOPE) was −17.124 (*p* < 0.001), indicating that the level of depression decreased after starting MMT. Taking the total improvement in the level of depression during the 9-month treatment duration as a whole, an 86% improvement was observed during the first 3 months of MMT, as indicated by a regression weight between the slope and the first 3-month follow-up level of depression of 0.86. The patients exhibited a total improvement in the level of depression of 91% at the second 3-month follow-up point during MMT, the regression weight between the slope and the second 3-month follow-up level of depression being 0.91. Meanwhile, the estimated mean of the intercept (ICEPT) was 28.873 (*p* < 0.001), and furthermore, the variances of the SLOPE and ICEPT were significant (*p* < 0.001).

The female heroin users had a higher level of depression at intake than the male heroin users, the regression weight from gender to ICEPT being significant and negative (coeff = −7.941, *p* < 0.001). In contrast, the level of depression of the female heroin users reduced more rapidly than that of the male heroin users, the regression weight from gender to SLOPE being significantly positive, which resulted in a less negative slope (coeff = 4.236, *p* < 0.001). In addition, the level of depression of the younger heroin users decreased faster than that of the older heroin users, age having a significantly positive regression weight on SLOPE (coeff = 0.178, *p* = 0.033). However, age did not have a significant effect on ICEPT (coeff = −0.001, *p* = 0.992). The methadone dosage did not have a significant effect on the change in the level of depression (coeff = −0.034, *p* = 0.064).

## Discussion

There were several interesting findings in this study. The first important finding was that the heroin users had a markedly high level of depression before starting MMT, the average CES-D score being higher than 15, with a previous study recommending that a total CES-D score of 15 or higher indicates the presence of clinically significant depression (22). Depression is a common comorbidity in people with substance user disorder (23). Furthermore, heroin users may have a higher level of depression than that of the general population, with up to one-quarter of users meeting the criteria for major depression (24). A previous study indicated that heroin users may have different prevalence rates of major depressive disorder at the initiation of different treatment modalities (7). Our results were in line with those of previous studies. Furthermore, this study showed that there were individual differences in the level of depression in patients undergoing the same treatment modality, as indicated by the statistically significant variance of the intercept. Previous studies have shown that depression is a high-risk factor for suicide among heroin users (25, 26). The findings of the present and previous studies indicated that depression is an important mental health issue for heroin users, and highlighted the importance of screening for depression in heroin users planning to undergo MMT.

A previous study of the general population demonstrated that females have a higher level of depression than males (27). Sordo et al. (18) demonstrated that female heroin users have a higher risk of developing major depression than male heroin users. Furthermore, Maremmani et al. (28) indicated that female heroin users had a higher prevalence of a current major depressive episode than male heroin users when they were about to enter into treatment. The findings of previous studies have provided some clues as to a gender difference in the level of depression at intake for MMT. Our results supported the results of previous studies indicating that female heroin users have a higher level of depression than male heroin users. Taking gender into consideration, the variance in the level of depressive symptoms at intake was still significant. This meant that not only gender, but also factors other than gender, may contribute to the heterogeneous level of depressive symptoms among heroin users. For example, psychosocial stressors, such as a negative life event, may be associated with severity of depression in heroin users (29).

Another interesting finding was that the level of depression improved in both the male and female heroin users undergoing MMT. Most of the improvement in depression was observed during the first 3 months after intake. A previous study of the short-term outcomes of heroin users undergoing MMT found that the severity of mental health problems had decreased at the end of a 3-month follow-up period (30). Our results were not only in line with those of that previous study, but also demonstrated that most of the improvement in depression manifested during the first 3 months of MMT. This result was important, because it implied that heroin users undergoing MMT require intervention to reduce their level of depression, as according to the trajectory of depression delineated in this study, the severity of depression after 3 months of MMT was not low, and that beyond the 3-month point did not change greatly.

Few studies have explored the gender effect on improvement of depression in heroin users undergoing MMT. The present study found that the level of depression in the female heroin users decreased more rapidly after receiving MMT than that in the male heroin users. Although the female heroin users had a higher level of depression at intake than the male subjects, the gender difference in the level of depression was not significant after 3 months of MMT. This meant that MMT can confer on female heroin users faster and greater improvement in the level of depression.

We also observed a positive significant effect of age on the change in the level of depression. Compared with the younger heroin users, the older heroin users had a slower rate of decrease in the level of depression. In addition, age is a risk factor for depression (19, 31). Therefore, clinicians may need to pay more attention to the issue of depression when treating older heroin users.

There were several limitations of this study. First, although the present study was a prospective observational study, the causality between depression and MMT still could not be exactly determined. In addition, we surveyed the severity of depression using self-reported instruments, and did not make clinical diagnoses using standard diagnostic interviewing procedures. Finally, it should be noted that this was a sample of active treatment seekers, all of whom underwent MMT for 9 months.

## Conclusion

This study demonstrated that female and male heroin users had a similar trajectory of the level of depression, most of the improvement being observed to have developed during the first 3 months of treatment, with only minimal improvement being seen beyond the 3-month point. Meanwhile, the severity of depression of the female heroin users improved more rapidly than that of the male heroin users. Irrespective of the improvement in the level of depression, the heroin users had a high incidence of depression at intake of MMT. Regular screening for depression in heroin users is warranted to ensure early identification of those with depression.

## Ethics Statement

The protocol was approved by the Institutional Review Board of Kaohsiung Medical University.

## Author Contributions

P-WW drafted the work; H-CL and Y-HY collected data; C-YH, K-SC, and H-CW analyzed the data; and CFY designed this study.

## Conflict of Interest Statement

The authors declare that the research was conducted in the absence of any commercial or financial relationships that could be construed as a potential conflict of interest.
